# Massive Localized Lymphedema: Two Case Studies and Diagnostic Challenges

**DOI:** 10.7759/cureus.47092

**Published:** 2023-10-16

**Authors:** Saleh A Ba-shammakh, Hasn M Haj-Freej, Yanof S Al-naggar, Waleed Ghanem, Manal Z Haij, Fahmi Al-Mohd, Eman Hijazi, Samir S Amr

**Affiliations:** 1 Department of General Surgery, Princess Rahma Teaching Hospital, Irbid, JOR; 2 Department of General Surgery, The Islamic Hospital, Amman, JOR; 3 Department of Pathology and Microbiology, The Islamic Hospital, Amman, JOR; 4 Department of Pathology and Laboratory Medicine, Istishari Hospital, Amman, JOR

**Keywords:** pathophysiology, secondary lymphedema, surgical resection, morbid obesity, mll, massive localized lymphedema

## Abstract

Massive localized lymphedema (MLL) is an emerging clinical phenomenon predominantly observed in morbidly obese individuals. It presents both diagnostic and therapeutic challenges to clinicians due to its characterization by large, pendulous masses in the abdomen or thigh. MLL may resemble malignant conditions, such as liposarcoma, leading to unnecessary invasive interventions. This study presents two case studies: a 74-year-old male who succumbed to postoperative complications and a 56-year-old female who experienced successful recovery. These cases highlight the urgent need for robust diagnostic criteria and evidence-based management approaches for MLL. In addition, further research exploring the pathogenesis, risk factors, and potential connections among MLL, hypothyroidism, and angiosarcoma is essential.

## Introduction

Massive localized lymphedema (MLL) is a distinctive condition predominantly found in individuals with severe obesity. The exact epidemiology of MLL remains unclear; however, the rising global rates of obesity suggest a potential increase in its prevalence [1-2]. MLL is linked to disruptions in efferent lymphatic flow, possibly due to the abundance of adipose tissue [3]. Other contributing factors to MLL may include prior surgery, injury, or hypothyroidism [4]. Despite these insights, the specific pathogenesis of MLL remains elusive. As such, diagnosing MLL is challenging from clinical, pathological, and radiological perspectives because the soft tissue lesions associated with it can be mistaken for cancerous growths. There have been documented cases of MLLs being surgically removed similarly to tumors due to their presentation as large masses that resemble liposarcoma or lymphosarcoma upon radiological and histological examinations [5-7]. This has led to MLL being referred to as a “pseudosarcoma” [8]. Moreover, some clinicians suggest that MLL may contribute to the onset of angiosarcoma [9-12]. Herein, we report two case studies highlighting the diagnostic and treatment challenges presented by MLL, underscoring the need for robust diagnostic criteria and effective management strategies for this condition.

## Case presentation

Case 1

A 74-year-old male, with a medical history of type II diabetes mellitus, hypertension, obstructive sleep apnea, and gout, presented to the outpatient clinic with a chief complaint of an enlarging pendulous abdominal mass that had been developing over four years. The mass limited his daily activities and self-hygiene routines without being associated with gastrointestinal symptoms or systemic changes.

During the initial physical examination, the patient was alert and oriented. His vital signs were as follows: temperature of 36.7°C, blood pressure of 121/60 mmHg, respiratory rate of 20 breaths per minute, pulse rate of 90 beats per minute, and oxygen saturation of 93% on room air. The patient weighed 140 kg and was 163 cm tall, with a body mass index (BMI) of 52.7 kg/m². Notably, a central, pendulous abdominal mass with a centrally shifted umbilicus and nodularity of the overlying skin was observed.

Preoperative preparations consisted of laboratory tests (Table 1), comprehensive cardiac and respiratory evaluations, administration of prescribed medications, and cleaning of the abdomen with chlorhexidine wash.

**Table 1:** Laboratory investigations WBC: white blood cells, HB: hemoglobin, PCV: packed cell volume, PT: prothrombin time, INR: international normalized ratio, HbA1C: hemoglobin A1C, BUN: blood urea nitrogen, Cr.: creatinine, Na: sodium, K: potassium

Test	Value	Reference Range
WBC	6.3	4.5-11 x 10^9^/L
HB	12.3	12-18 g/dL
PCV	40	38-50%
Platelets	144	150-450 x 10^9^/L
PT	16	11-13.5 seconds
INR	1.2	0.8-1.1
HbA1C	12.5	4-5.6%
BUN	46	15-50 mg/dL
Cr.	1.23	0.84-1.21 mg/dL
Na	134	135-145 mmol/L
K	4.1	3.5-5.1 mmol/L

**Figure 1:**
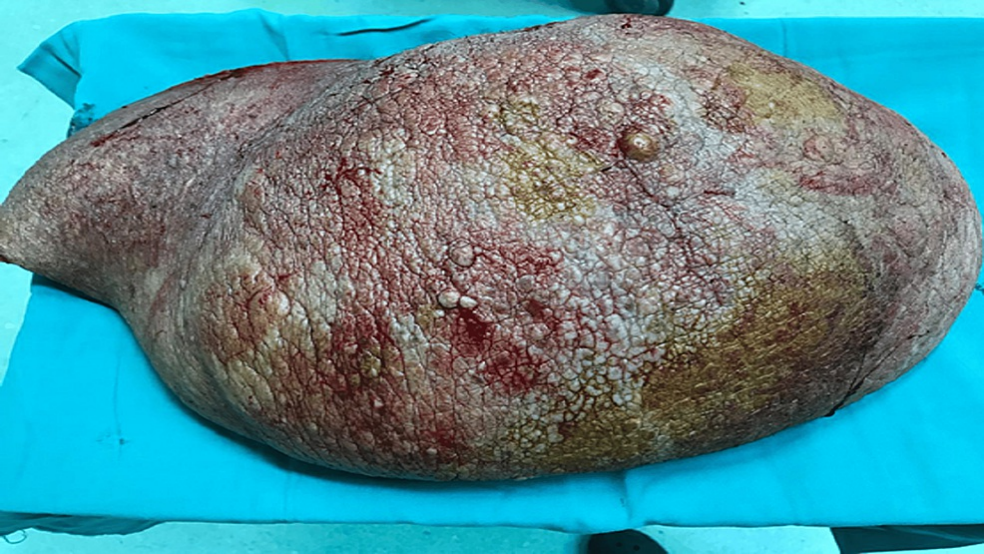
Post-surgical view of the excised abdominal mass

The patient underwent an excision of the abdominal mass under general anesthesia; the mass weighed approximately 6 kg (Figure 1).

**Figure 2:**
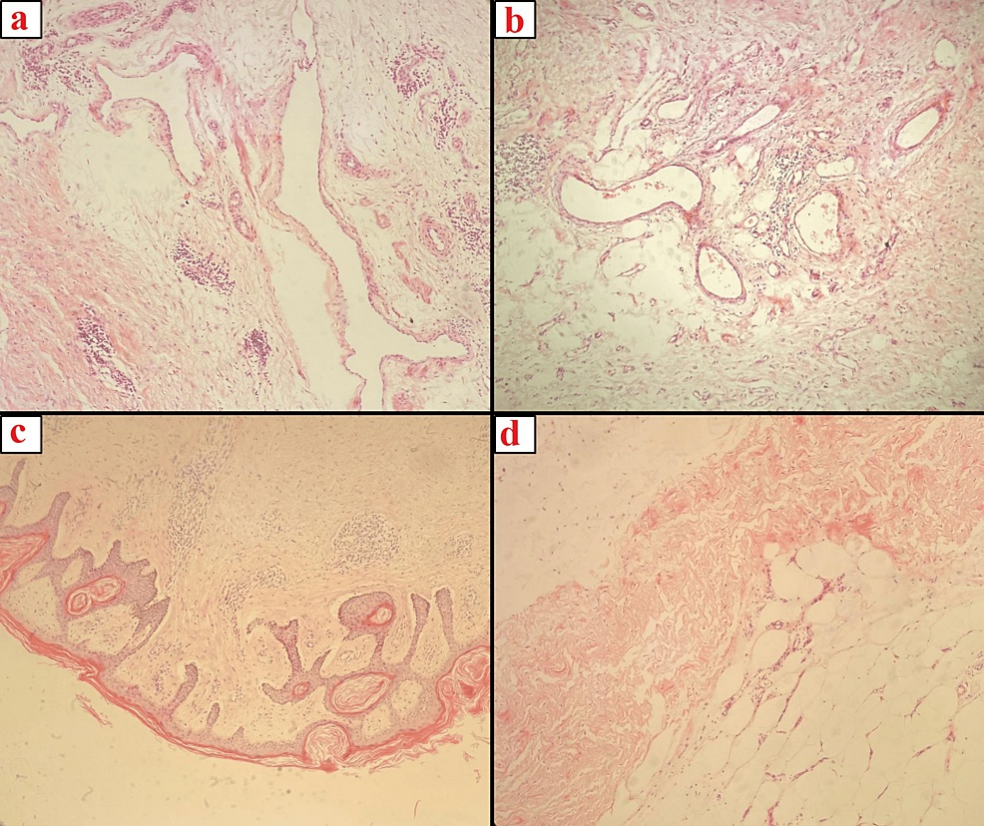
Histopathological findings a, b: Aggregates of irregular dilated lymphatic channels; hemotoxylin and eosin (H&E) stain, x200. c: Thickened papillomatous epidermis; H&E stain, x 100. d: Edematous thick fibrous bands intersecting adipose tissue; H&E stain, x 200.

Two abdominal R-VAC drains were placed during surgery. Pathology reports confirmed the diagnosis of MLL, characterized by overgrowth of skin and edematous adipose tissue in the underlying subcutaneous layer (Figure 2).

At day one post-op, the patient's course was complicated by an increase in creatinine levels from 1.2 to 1.6 mg/dL (normal range: 0.7 to 1.3 mg/dL), leading to his admission to the intensive care unit (ICU). Unfortunately, his condition deteriorated due to the development of pneumonia, necessitating intubation. The patient’s health declined continuously over the next three days, culminating in his demise.

Case 2

A 56-year-old morbidly obese female presented to the clinic with a seven-year history of a progressively enlarging mass on her right thigh. Initially painless, the mass had significantly enlarged in recent years, affecting her quality of life and daily activities. The overlying skin exhibited induration and a brownish discoloration. Her medical history included hypertension and morbid obesity, with current medications comprising statins, aspirin, beta-blockers, amlodipine, and valsartan.

Upon examination, the patient was talkative, oriented, and not in visible distress. The recorded vital signs included a temperature of 36.9°C, blood pressure of 122/66 mmHg, respiratory rate of 20 breaths per minute, pulse of 109 beats per minute, and oxygen saturation of 92% on room air. The patient’s weight was 149 kg (height not recorded). Examination of the right lower extremity revealed a 15 cm x 20 cm pedunculated mass with overlying brown, indurated, and thickened skin.

**Figure 3:**
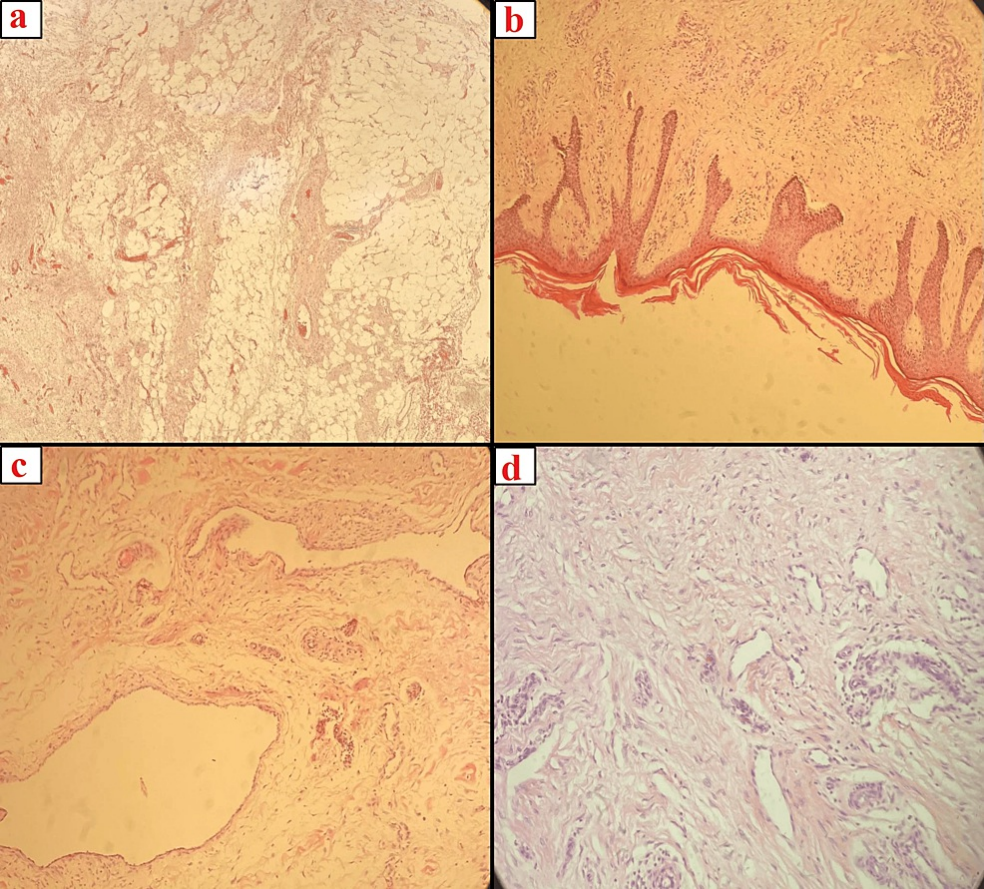
Histopathological findings a: Thick fibrous bands alternating with edematous adipose tissue; hemotoxylin and eosin (H&E) stain, x40. b: Thickened skin with dermal fibrosis; H&E stain, x100. c, d: Irregular ectatic lymphatic spaces; H&E stain, x200.

The patient underwent excision of the mass under spinal anesthesia. The excised mass weighed approximately 7 kg. A negative pressure drain was inserted during the procedure. The pathology report aligned with the clinical diagnosis, revealing hypertrophied papillomatous epidermis and edematous fatty tissue in the underlying subcutaneous layer (Figure 3).

Postoperatively, the patient remained stable and recovered successfully without complications. She was discharged five days later, and follow-up assessments at six and 12 months showed no signs of complications or recurrence of the disease.

## Discussion

MLL is recognized as a distinct clinical entity, initially delineated by Farshid and Weiss in 1998 [5], who described 14 cases of MLL occurring exclusively in morbidly obese individuals [5]. Notably, our case studies align with these findings, as both patients were morbidly obese, thereby adding empirical weight to the existing literature [3-4,8,13-14]. With escalating global obesity rates, there is renewed interest in the MLL phenomenon [1-2]. While the precise prevalence remains uncertain, studies suggest that women are more commonly affected than men [15-16].

MLL predominantly manifests in individuals with a BMI exceeding 40 kg/m², classified as a secondary form of lymphedema [17]. Likely resulting from disruptions in lymphatic drainage due to extreme obesity [18], our first patient also had comorbidities, such as type II diabetes mellitus and hypertension. However, existing literature only suggests a possible link between MLL and hypothyroidism, which warrants further research [4,19].

MLL is typically characterized by large, drooping masses, often located in the thighs or the abdominal region, as observed in our first case [3,5]. Initially, the condition presents as soft, non-painful pitting edema; however, as it progresses, the tissue hardens and loses its pitting quality, as evidenced in our second case where the overlying skin became indurated and discolored [20]. Histopathologically, the features in our cases were consistent with those of MLL as previously described by Farshid and Weiss. These features include mild hyperkeratosis of the epidermis, thickening of the overlying skin, and the presence of abundant fat lobules within the dermis that are separated by connective tissue septa [5]. The septa are composed of collagen fibrils, edematous fluid, and fibroblasts. In addition, capillaries and small arterioles frequently proliferate at the boundary between the fat lobules and connective tissue septa [5]. No dysplastic features, such as hyperchromasia or nuclear pleomorphism, were observed in any of the cases [5]. Histological assessments confirmed typical MLL features, such as extensive dermal fibrosis and dilated lymphatic channels, without muscle involvement [5,21-23]. Our findings underline the potential for MLL to be misdiagnosed, given its similarities to other conditions [24-25].

The MLL treatment strategy depends on the location, severity, and impact of the mass on functionality. Both patients in our case studies underwent surgical resection. The first case experienced postoperative complications, underscoring the need for meticulous preoperative preparation and continuous monitoring [26-27]. The successful recovery of the second patient highlights the necessity of comprehensive management plans, including controlled caloric intake and graded physical activity, to prevent recurrence [28].

MLL is generally benign, but neglecting treatment can result in transformation into angiosarcoma or Stewart-Treves syndrome [16]. This fact is especially relevant to our first case, where the patient's demise due to postoperative complications underscores the disease's seriousness and potential escalation into life-threatening conditions, with some cases having a mortality rate of up to 9% [29].

## Conclusions

Although the understanding of MLL has grown since its identification, diagnostic challenges remain due to its mimicry of malignant conditions. The presented cases exemplify the typical MLL demographic and showcase different outcomes. Early diagnosis, personalized management plans, and rigorous postoperative care are crucial to mitigate risks and improve prognosis. As global obesity rates rise, understanding MLL becomes increasingly vital. Future research should focus on clarifying its pathophysiology, evaluating nonsurgical interventions, and exploring links to potential comorbidities, including angiosarcoma.

## References

[1] (2016). Trends in adult body-mass index in 200 countries from 1975 to 2014: a pooled analysis of 1698 population-based measurement studies with 19·2 million participants. Lancet.

[2] (2017). Worldwide trends in body-mass index, underweight, overweight, and obesity from 1975 to 2016: a pooled analysis of 2416 population-based measurement studies in 128·9 million children, adolescents, and adults. Lancet.

[3] Manduch M, Oliveira AM, Nascimento AG, Folpe AL (2009). Massive localised lymphoedema: a clinicopathological study of 22 cases and review of the literature. J Clin Pathol.

[4] Wu D, Gibbs J, Corral D, Intengan M, Brooks JJ (2000). Massive localized lymphedema: additional locations and association with hypothyroidism. Hum Pathol.

[5] Farshid G, Weiss SW (1998). Massive localized lymphedema in the morbidly obese: a histologically distinct reactive lesion simulating liposarcoma. Am J Surg Pathol.

[6] Porrino J, Walsh J (2016). Massive localized lymphedema of the thigh mimicking liposarcoma. Radiol Case Rep.

[7] Kotidis E, Cepaityte D, Petrakis G, Sapalidis K, Kanellos I (2015). Massive localized lymphedema in the morbidly obese patient: a clinical entity mimicking lymphosarcoma. Wounds.

[8] Goshtasby P, Dawson J, Agarwal N (2006). Pseudosarcoma: massive localized lymphedema of the morbidly obese. Obes Surg.

[9] Salas S, Stock N, Stoeckle E, Kind M, Bui B, Coindre JM (2008). Chronic lymphedema due to morbid obesity: an exceptional cause of abdominal wall angiosarcoma. Virchows Arch.

[10] Azam M, Saboorian H, Bieligk S, Smith T, Molberg K (2001). Cutaneous angiosarcoma complicating morbid obesity. Arch Pathol Lab Med.

[11] Shehan JM, Ahmed I (2006). Angiosarcoma arising in a lymphedematous abdominal pannus with histologic features reminiscent of Kaposi's sarcoma: report of a case and review of the literature. Int J Dermatol.

[12] Shon W, Ida CM, Boland-Froemming JM, Rose PS, Folpe A (2011). Cutaneous angiosarcoma arising in massive localized lymphedema of the morbidly obese: a report of five cases and review of the literature. J Cutan Pathol.

[13] Wang NS, Walters RF, Warren SJ (2010). Massive localized lymphedema: a soft tissue process that may present to dermatologists. Am J Dermatopathol.

[14] Modolin ML, Cintra W Jr, Paggiaro AO, Faintuch J, Gemperli R, Ferreira MC (2006). Massive localized lymphedema (MLL) in bariatric candidates. Obes Surg.

[15] Evans RJ, Scilley C (2011). Massive localized lymphedema: a case series and literature review. Can J Plast Surg.

[16] Chopra K, Tadisina KK, Brewer M, Holton LH, Banda AK, Singh DP (2015). Massive localized lymphedema revisited: a quickly rising complication of the obesity epidemic. Ann Plast Surg.

[17] Bennett JM, Mehta S, Rhodes M (2007). Surgery for morbid obesity. Postgrad Med J.

[18] Warren AG, Brorson H, Borud LJ, Slavin SA (2007). Lymphedema: a comprehensive review. Ann Plast Surg.

[19] Iurkevich NP, Savchenko TV (1994). Lymphedema and hypothyroidism [Article in Russian]. Klin Med (Mosk).

[20] Lobato RC, Zatz RF, Cintra Junior W, Modolin ML, Chi A, Van Dunem Filipe de Almeida YK, Gemperli R (2019). Surgical treatment of a penoscrotal massive localized lymphedema: case report. Int J Surg Case Rep.

[21] Asch S, James WD, Castelo-Soccio L (2008). Massive localized lymphedema: an emerging dermatologic complication of obesity. J Am Acad Dermatol.

[22] Vives F, García-Perdomo HA, Ocampo-Flórez GM (2016). Giant lymphedema of the penis and scrotum: a case report. Autops Case Rep.

[23] Fife CE, Carter MJ (2008). Lymphedema in the morbidly obese patient: unique challenges in a unique population. Ostomy Wound Manage.

[24] Fadare O, Brannan SM, Arin-Silasi D, Parkash V (2011). Localized lymphedema of the vulva: a clinicopathologic study of 2 cases and a review of the literature. Int J Gynecol Pathol.

[25] D'Antonio A, Caleo A, Boscaino A, Mossetti G, Iannantuoni N (2010). Vulvar lymphoedematous pseudotumours mistaken for aggressive angiomyxoma: report of two cases. Gynecol Obstet Invest.

[26] Moon Y, Pyon JK (2016). A rare case of massive localized lymphedema in a morbidly obese patient. Arch Plast Surg.

[27] Heller DS, Fitzhugh VA, Cracchiolo B, Barrett T Jr, Suidan RS (2014). Massive localized lymphedema of the vulva: report of a case and review of the literature. J Low Genit Tract Dis.

[28] Bahrami A, Ronaghan JE, O-Yurvati AH (2015). Pseudosarcoma of the thigh: a rare case of massive localized lymphedema. Int Surg.

[29] Fife C (2014). Massive localized lymphedema, a disease unique to the morbidly obese: a case study. Ostomy Wound Manage.

